# Speech discrimination performance in multiple sclerosis dataset

**DOI:** 10.1016/j.dib.2020.106614

**Published:** 2020-12-03

**Authors:** Pippa Iva, Joanne Fielding, Meaghan Clough, Owen White, Gustavo Noffs, Branislava Godic, Russell Martin, Anneke van der Walt, Ramesh Rajan

**Affiliations:** a Neuroscience Discovery Program, Biomedicine Discovery Institute, Department of Physiology, Monash University, Melbourne, Australia; b Department of Neurosciences, Central Clinical School, Alfred Hospital, Monash University, Melbourne, Australia; c Centre for Neuroscience of Speech, University of Melbourne, Melbourne, Australia

**Keywords:** Multiple sclerosis, Speech discrimination, Central auditory processing, Audiometry, Psychoacoustics, Auditory perception, Biomarkers

## Abstract

The most complex interactions between human beings occur through speech, and often in the presence of background noise. Understanding speech in noisy environments requires the integrity of highly integrated and widespread auditory networks likely to be impacted by multiple sclerosis (MS) related neurogenic injury. Despite the impact auditory communication has on a person's ability to navigate the world, build relationships, and maintain employability; studies of speech-in-noise (SiN) perception in people with MS (pwMS) have been minimal to date. Thus, this paper presents a dataset related to the acquisition of pure-tone thresholds, SiN performance and questionnaire responses in age-matched controls and pwMS. Bilateral pure-tone hearing thresholds were obtained at frequencies of 250 hertz (Hz), 500 Hz, 750 Hz, 1000 Hz, 1500 Hz, 2000 Hz, 4000 Hz, 6000 Hz and 8000 Hz, and hearing thresholds were defined as the lowest level at which the tone was perceived 50% of the time. Thresholds at 500 Hz, 1000 Hz, 2000 Hz and 4000 Hz were used to calculate the four-tone average for each participant, and only those with a bilateral four tone average of ≤ 25 dB HL were included in the analysis. To investigate SiN performance in pwMS, pre-recorded Bamford-Kowal-Bench (BKB) sentences were presented binaurally through headphones at five signal-to-noise ratios (SNR) in two noise conditions: speech-weighted noise and multi-talker babble. Participants were required to verbally repeat each sentence they had just heard; or indicate their inability to do so. A 33-item questionnaire, based on validated inventories for specific adult clinical populations with abnormal auditory processing, was used to evaluate auditory processing in daily life for pwMS. For analysis, pwMS were grouped according to their Expanded Disability Status Scale (EDSS) score as rated by a neurologist. PwMS with EDSS scores ≤ 1.5 were classified as ‘mild’ (*n* = 20); between 2 and 4.5 as ‘moderate’ (*n* = 16) and between 5 and 7 as ‘advanced’ (*n* = 10) and were compared to neurologically healthy controls (*n* = 38). The outcomes of the SiN task conducted in pwMS can be found in Iva et al., (2021). The present data has important implications for the timing and delivery of preparatory education to patients, family, and caregivers about communication abilities in pwMS. This dataset will also be valuable for the reuse/reanalysis required for future investigations into the clinical utility of SiN tasks to monitor disease progression.

## Specifications Table

**Table:** 

Subject	Biological Sciences
Specific subject area	Speech discrimination in noise in people with multiple sclerosis
Type of data	Table
	Graph
How data were acquired	Audiometric examinations were conducted using a Beltone Model 110 Clinical Audiometer and calibrated TDH headphones.
	SiN discrimination tasks were presented to participants binaurally through Sennheiser HD535 headphones, driven by a Dell Latitude computer.
	Stimuli were calibrated by coupling the headphones to a Brüel and Kjaer Artificial Ear Type 4152 containing a Brüel and Kjaer 1 Condenser Microphone Type 4145. The microphone output was connected to a Brüel and Kjaer Precision Sound Level Meter Type 2203 from which sound pressure level (SPL) was read directly.
	Subjective responses were collected using a questionnaire based on validated inventories for specific adult clinical populations with abnormal auditory processing
Data format	Raw
	Analyzed
Parameters for data collection	Data collection was obtained if participants had a definite diagnosis of MS [1]; were aged between 18 and 65 years; had no other neurological disease; and no MS relapse or treatment with corticosteroids in the three months prior to auditory testing
Description of data collection	The data was collected between May 2017 to August 2019. Tests were conducted over two, one-hour sessions (no greater than one month apart), with intermittent breaks whenever requested by the participants. All auditory testing was conducted in a quiet room with minimal distractions. Data was stored using an in-house program.
Data source location	Monash University
	Clayton Campus, Victoria
	Australia
Data accessibility	With the article
Related research article	P. Iva, J. Fielding, M. Clough, O. White, G. Noffs, B. Godic, R. Martin, A. van der Walt, R. Rajan, Speech discrimination impairments as a marker of disease severity in multiple sclerosis. Multiple Sclerosis and Related Disorders. 2021;47.
	https://doi.org/10.1016/j.msard.2020.102608

## Value of the Data

• This dataset advances the knowledge of the impact of MS on understanding speech in background noise - a common occurrence in everyday life.
• The present data is particularly useful for clinicians, healthcare workers and researchers to garner an understanding of the impact that MS may have on auditory communication, and thereby inform a more holistic approach to treatment and care.
• The present data also has important implications for the timing and delivery of preparatory education to patients, family, and caregivers about potential changes in communication abilities which may worsen with disease progression.
• This dataset can be reanalyzed and reused for future investigations into the clinical utility of SiN tasks to monitor disease progression in MS, as SiN tasks have the advantages of being fast, cost effective, easy to administer, and non-invasive.

## 1 Data Description

### 1.1 Audiometry

**Fig. 1:**
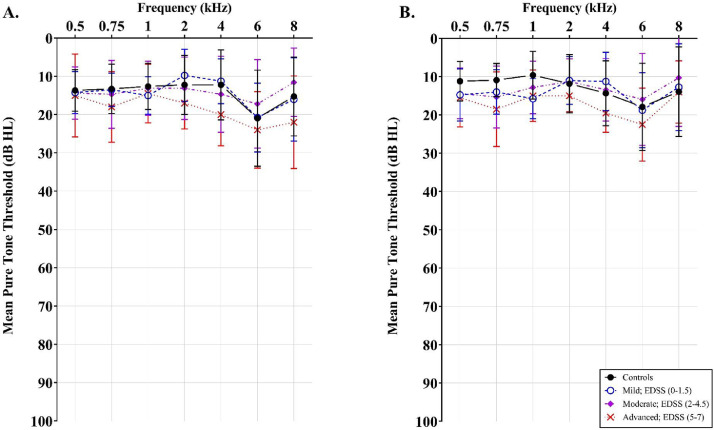
Mean pure tone thresholds (dB HL) ± standard error of the mean (SEM) obtained for left (A) and right (B) ears in controls (black circle; *n* = 38) and pwMS with mild (blue, open circle; *n* = 20), moderate (purple diamond; *n* = 16) and advanced (red cross; *n* = 10) disability. No statistical differences in mean pure tone thresholds were found between groups in the left or right ear (two-way ANOVA). (For interpretation of the references to color in this figure legend, the reader is referred to the web version of this article.)

This dataset contains raw and processed data of pure-tone thresholds obtained from controls and pwMS. Supplementary Table 1 presents the raw data of all participants who were tested, recorded as decibels Hearing Level (dB HL) relative to normal sensitivity [2] at all frequencies tested. Fig. 1 displays the mean ± SEM pure tone thresholds (dB HL) obtained for the left (A) and right (B) ears of controls and pwMS with bilateral pure-tone averages of ≤ 25 dB HL only. A two-way analysis of variance (ANOVA) using IBM Statistical Package for the Social Sciences (SPSS) version 26 was conducted to compare the mean pure-tone averages of the three MS disability groups and controls. No significant difference was found between controls, mild, moderate and advanced pwMS in both the left [F (3,80) = 2.66, *p* = 0.06, ƞ² = 2.59] and right ears [F(3,80) = 2.36, *p* = 0.08, ƞ² = 2.51]. The audiometric results of controls and pwMS are discussed in the related research article [3].

### 1.2 SiN discrimination

This dataset contains raw and processed data of SiN discrimination performance obtained from controls and pwMS. Supplementary Table 2 presents the raw data of responses (correct/incorrect) made by all participants at every trial (10 at each SNR), at 5 SNRs, in both speech-weighted and multi-talker babble noise (i.e. each participant provided 100 observations, with the exception of a few who did not complete the full test battery). For analysis, the correct responses at each SNR (out of 10) were totaled for each noise masker type. Boltzmann sigmoidal functions using Graphpad Prism 8 were fitted to this data to obtain psychometric curves as a function of SNR for individual participants. The top and bottom of the functions were constrained to 10 and 0 sentences correct, respectively. Measures of goodness of fit were strong for each group (R² always ≥ 0.89).

Slope (sentences/dB) and midpoint data (dB) from the curves were extracted and the mean averages across controls and MS disability groups were compared using a one-way ANOVA (refer to Tables 1 & 2). There was no significant difference in slopes (sentences/dB) between the listening groups in speech-weighted noise [F(3,77)=1.70,*p* = 0.18] and babble [F(3,80)=0.3,*p* = 0.83]. In contrast, the midpoints of the curves were significantly different amongst listener groups in speech-weighted noise [F(3,77)=7.48,*p* = 0.0002] and babble [F(3,80) = 14.84,*p*<0.0001]. The outcomes of the SiN tasks conducted in pwMS are discussed in Iva et al., 2021.

**Table 1:** Goodness of fit (*R*²), midpoint and slope values (± SE) for Boltzmann sigmoidal functions for the performance of controls and MS participants with mild, moderate, and advanced impairment in the sentences in speech-weighted noise task.

		Midpoint ± SE	Slope ± SE
	*R*²	(dB)	(sentence/dB)
**Controls**	0.95	−6.79 ± 0.19	1.54 ± 0.13
**MS; Mild disability**			
(EDSS 0–1.5; Median = 0)	0.93	−6.38 ± 0.22	1.55 ± 0.15
**MS; Moderate disability**			
(EDSS 2–4.5; Median = 2.5)	0.92	−5.86 ± 0.31*	1.96 ± 0.14
**MS; Advanced disability**			
(EDSS 5–7; Median = 6)	0.89	−4.85 ± 0.43****	1.92 ± 0.2

* (*p* < 0.05); **** (*p*< 0.0001) compared to controls (One Way ANOVA, Tukey's post hoc test) The top and bottom of the Boltzmann sigmoidal functions were constrained to 10 and 0 respectively.

**Table 2:** Goodness of fit (*R*²), midpoint and slope values (± SE) for Boltzmann sigmoidal functions for the performance of controls and MS participants with mild, moderate and advanced impairment in the sentences in babble task.

	*R*²	Midpoint ± SE	Slope ± SE
		(dB)	(sentence/dB)
**Controls**	0.95	−0.39 ± 0.38	1.43 ± 0.08
**MS; Mild disability**			
(EDSS 0–1.5; Median = 0)	0.94	0.27 ± 0.2*	1.57 ± 0.11
**MS; Moderate disability**			
(EDSS 2–4.5; Median = 2.5)	0.92	0.75 ± 0.2**	1.46 ± 0.13
**MS; Advanced disability**			
(EDSS 5–7; Median = 6)	0.94	1.45 ± 0.31***	1.42 ± 0.25

* (*p* < 0.05); ** (*p* < 0.001) **** (*p* < 0.0001) compared to controls (One Way ANOVA, Tukey's post hoc test) The top and bottom of the Boltzmann sigmoidal functions were constrained to 10 and 0 respectively.

### 1.3 Auditory attention and discomfort questionnaire (AADQ)

This dataset contains the AADQ responses obtained from controls and pwMS. Supplementary Table 1 presents the raw data of all participants who were tested, recorded as a score on a seven-point Likert scale; 1 indicated strong disagreement and 7 indicated strong agreement with each statement (33 items in total). A copy of the questionnaire, as well as the outcomes of the responses from controls and pwMS are presented and discussed in Iva et al., 2021.

## 2 Experimental Design, Materials and Methods

### 2.2 Participants

Thirty-eight controls were recruited from the local community, and forty-six people with confirmed MS by revised McDonald criteria [1] were recruited through the Royal Melbourne Hospital Australia. Participants were excluded if they had a hearing loss (refer to Section 2.4 Audiometry for the definition of hearing loss) and no pwMS experienced recent (within 30 days) relapses and/or steroids administration. All participants reported English as their native language and provided informed written consent.

PwMS were grouped according to EDSS score [4] as rated by a neurostatus certified neurologist. PwMS with EDSS scores ≤ 1.5 were classified as ‘mild’; between 2 and 4.5 as ‘moderate’ and between 5 and 7 as ‘advanced’ disability.

### 2.3 Overview

All participants completed a wide-ranging assessment battery which included standard audiometric evaluations and speech discrimination tasks. These tests were conducted over two, one-hour sessions (no greater than one month apart), with intermittent breaks whenever requested by the participants. Testing locations were at Monash University Clayton Campus, Royal Melbourne Hospital, or private residences. All auditory testing was conducted in a quiet room with minimal distractions.

### 2.4 Audiometry

The hearing status of all participants was determined using pure tone audiometry with a Beltone Model 110 Clinical Audiometer and calibrated TDH headphones. Sensitivity was tested one ear at a time, at standard audiometric frequencies of 250 hertz (Hz), 500 Hz, 750 Hz, 1000 Hz, 1500 Hz, 2000 Hz, 4000 Hz, 6000 Hz, and 8000 Hz, using a modified Hughson-Westlake procedure [5]. Hearing thresholds, recorded as decibels Hearing Level (dB HL) relative to normal sensitivity [2], were defined as the lowest level at which the tone was perceived 50% of the time. Only subjects with a bilateral PTA (measured at 500 Hz, 1000 Hz, 2000 Hz and 4000 Hz) of ≤25 dB HL were used for the analysis of the SiN dataset.

### 2.5 SiN discrimination tasks

SIN discrimination tasks were conducted from a Dell Latitude computer, using an in-house program (designed by Dr Chris James formerly of the Bionic Ear Institute, University of Melbourne), to deliver the sentences and noise at varying SNRs and to store and display data. All auditory stimuli were stored as “.wav” files and presented to participants binaurally through Sennheiser HD535 headphones. For every trial, target sentences were always presented at 70 dBA, an arbitrary level for comfortable listening (https://www.chem.purdue.edu/chemsafety/Training/PPETrain/dblevels.htm), whilst maskers were presented at a range of levels to generate different SNRs.

Stimuli were calibrated by coupling the headphones to a Brüel and Kjaer Artificial Ear Type 4152 containing a Brüel and Kjaer 1 Condenser Microphone Type 4145. The microphone output was connected to a Brüel and Kjaer Precision Sound Level Meter Type 2203 from which sound pressure level (SPL) was read directly on an A-weighted scale on ‘slow’ time setting. Sentence levels were calibrated using a reference 1–15 kHz noise band signal with average root mean square level set to the same value as that for the sentences. The noise masker was calibrated by playing the noise through the headphones and using the “slow” time setting to measure output level.

#### 2.5.1 Target sentences

Sentences came from the Bamford–Kowal–Bench (BKB) sentence lists for partially-hearing children. The full BKB list contains 192 sentences, each 4–6 words long, with each sentence having three keywords by which identification of the sentence was scored [6].

Previous, unpublished work in our laboratory determined psychometric functions for the identification of each of the 192 BKB sentences in speech-weighted noise (i.e., noise shaped to have energy spread over frequencies as it is for speech). 15 normal-hearing participants were tested for the identification of each sentence at a number of SNRs and the SNR at which each fixed-intensity sentence was correctly discriminated 50% of the time was defined as the speech reception threshold (SRT). This SRT was the basis for the selection of 120 sentences with similar identifiability [7] and these sentences were then tested and validated against speech-weighted-noise and multi-talker-babble maskers in a large normal-hearing population of different age ranges segregated into decade age groups [8]. All target sentences were spoken by a female voice with an Australian accent in a neutral tone.

#### 2.5.2 Masker noise

Two background noises: 1) speech-weighted noise and 2) multi-talker babble were presented to all participants. Speech-weighted noise was shaped to the long-term average spectrum of the target sentences, as measured using a Madsen audiometer. Multi-talker babble consisted of eight simultaneous voices generated by doubling over and temporally offsetting a recording of four people reading nonsense text. Both noises were digitized and stored as .wav files. The root mean square levels of the two noises were modified to be equal.

#### 2.5.3 SiN test procedures

Unique sentences were presented one at a time with a background masker and participants were required to identify and verbally repeat each sentence they had just heard; or indicate if they were unable to do so. The experimenter recorded the responses and scored the correct/incorrect identification of the sentence using the in-house program, and then presented the next sentence after a 1.5 second delay. Sentences were scored as ‘correct’ when all three keywords were correctly identified. No time limit was placed on the response and feedback was not provided.

Sentences were presented at a constant volume whilst the masker level was varied to generate SNRs of 1,−1,−3,−5, and −7 dB in speech-weighed noise and 3, 1, −1, −3, and −5 dB in multi-talker babble. Prior to each noise condition, participants completed ten practice trials (ten unique target sentences) at a high SNR of +5 dB for acclimatization to stimuli. Subsequent SNR blocks were presented in random order. Ten unique sentences were presented at each SNR in a randomised order presentation.

### 2.6 AADQ

The AADQ was developed by William Dunlop, Peter Enticott and Ramesh Rajan (2016) and based on the following validated inventories for specific adult clinical populations experiencing abnormal auditory processing: the Hearing Handicap and Denver Scales [9], the Hearing Handicap Inventory for the Elderly [10], the Amsterdam Inventory for Auditory Disability and Handicap [11] and an unpublished inventory developed at The University of Auckland for hearing aid users.

The 33-item AADQ consisted of statements grouped into three components based on a Principal Components Analysis conducted on control data [12]. Component 1, the Audio-Attentional Difficulty subscale, measured difficulties attending to speech in noisy environments from fourteen items (items 18–30, 33). Component 2, the Auditory Discomfort (Non-Verbal) subscale, measured discomfort to non-verbal environmental sounds from eight items (items 1, 2, 4, 9–12, 14). Component 3, the Auditory Discomfort (Verbal) subscale, measured discomfort to verbal sounds from seven items (items 3, 6, 13, 15, 16, 31, 32). Subscale scores were generated by summing the response to each item that loaded onto it. Audio-Attentional Difficulty had a possible range of 14–98, Auditory Discomfort (Non-Verbal) had a possible range of 8–56, and Auditory Discomfort (Verbal) had a possible range of 5–35. Items 5, 7, 8 and 17 did not load onto any component and were hence excluded.

The questionnaire was completed on paper during the testing session under no time restriction. Participants responded on a seven-point Likert scale with 1 indicating strong disagreement and 7 indicating strong agreement with the statement. Participant scores were reversed for items 8, 17 and 31 as they had negative valence, (e.g., “When I am in a crowded supermarket talking with the cashier, I can follow the conversation”). For a description of all items on the questionnaire, refer to the related research article [3].

## Ethics Statement

All procedures were approved by the Monash University Human Research Ethics Committee (8170) and conformed to the guidelines of the National Health and Medical Research Council of Australia and the protocols of the Helsinki Declaration for experiments involving human participants. Prior to any experimentation, all subjects were provided with a plain-language explanatory statement and completed a consent form to confirm they were informed that their information would remain anonymous and that they were free to withdraw at any stage of testing.

## CRediT authorship contribution statement

**Pippa Iva:** Formal analysis, Investigation, Data curation, Project administration, Writing - review & editing. **Joanne Fielding:** Writing - review & editing. **Meaghan Clough:** Writing - review & editing. **Owen White:** Writing - review & editing. **Gustavo Noffs:** Investigation. **Branislava Godic:** Investigation, Writing - review & editing. **Russell Martin:** Supervision, Writing - review & editing. **Anneke van der Walt:** Investigation, Writing - review & editing. **Ramesh Rajan:** Conceptualization, Methodology, Software, Validation, Resources, Supervision, Writing - review & editing.

## Declaration of Competing Interest

This research was supported by internal lab funds at Monash University, Clayton, VIC, Australia. Dr Anneke van der Walt has received travel support and served on advisory boards for Novartis, Biogen, Merck Serono and Roche. She receives research support from the National Health and Medical Research Council of Australia and MS Research Australia. Dr Joanne Fielding has received funding for MS research from Genzyme, Biogen, and Novartis.

## Appendix Supplementary Materials

Image, application 1

Image, application 2

## Footnotes

Supplementary material associated with this article can be found in the online version at doi:10.1016/j.dib.2020.106614.
